# The Hidden Tragedy of Rivers: A Decade of Unintentional Fatal Drowning in Australia

**DOI:** 10.1371/journal.pone.0160709

**Published:** 2016-08-12

**Authors:** Amy E. Peden, Richard C. Franklin, Peter A. Leggat

**Affiliations:** 1 Royal Life Saving Society–Australia, Sydney, New South Wales, Australia; 2 School of Public Health and Tropical Medicine, College of Public Health, Medical and Veterinary Sciences, James Cook University, Townsville, Queensland, Australia; 3 Research School of Population Health, College of Medicine, Biology and Environment, Australian National University, Canberra, Australia; Leibniz Institute for Prevention Research and Epidemiology (BIPS), GERMANY

## Abstract

**Objective(s):**

Describe unintentional drowning deaths in rivers, creeks and streams (rivers) in Australia and identify risk factors to inform prevention.

**Design & Setting:**

This study is a cross-sectional, total population audit of all unintentional fatal drownings in Australian rivers between 1-July-2002 and 30-June-2012 using Australian coronial data. A modified Bonferroni test has been applied, deeming statistical significance p<0.03 and p<0.04 respectively.

**Results:**

Rivers (n = 770; 26.6%) were the leading location among the 2,892 people who died from unintentional fatal drowning. This is a rate of 0.37/100,000 people / annum. Within river drowning deaths common groups include; males (80.4%), adults (85.3%), adults who have consumed alcohol (25.5%), people who fell in (21.3%), people involved in non-aquatic transport incidents (18.2%) and locals (74.0%). Children were 1.75 times more likely than adults (p<0.04) to drown in rivers as a result of a fall and adults 1.50 times more likely to drown in rivers as a result of watercraft incidents when compared to children. When compared to males, females were 2.27 and 4.45 times respectively more likely to drown in rivers as a result of incidents involving non-aquatic transport (p<0.04) and being swept away by floodwaters (p<0.04). Males were 2.66 and 4.27 times respectively more likely to drown in rivers as a result of watercraft incidents (p<0.04) and as a result of jumping in (p<0.04) when compared to females.

**Conclusion(s):**

While rivers are the leading location for drowning in Australia, little is understood about the risks. This study has identified key groups (males, adults, locals) and activities. While males were more likely to drown, the risk profile for females differed.

## Introduction

Drowning is a neglected public health issue, with an estimated 372,000 lives lost worldwide per annum [1]. The majority occur in low and middle income countries as a result of activities associated with daily life [2]. This is in contrast to drowning deaths in high income countries which generally occur recreationally [1].

Until now drowning prevention research has focused on young children (particularly those under five). This has been successful in reducing drowning deaths of young children in private swimming pools predominantly through improved pool fencing [3]. In contrast, a systematic review of literature published between 1980 and 2014 found there had been little research that specifically focused on drowning in rivers, creeks and streams (henceforth referred to as rivers), nor is the profile of river drowning victims well understood [4]. In studies published to date internationally, the crude death rate per 100,000 people for fatal river drowning varied from a low of 0.20 to a high of 1.89 [4].

Since the completion of the systematic literature review, the World Health Organization (WHO) has published a global report on drowning, identifying it as a world-wide issue [1]. Subsequent to the release of this report, studies have continued to be published on the issue of drowning prevention [5–6]. Little research has been published on the issue of drowning in rivers, however several papers identify natural waterways (which includes rivers) as common drowning locations [7–9]. One of these studies modelled unintentional drowning mortality rates in Thailand (2000–2009), finding most drowning deaths occurred in rural areas, however did not explore specific drowning locations [7]. A Chinese study exploring drowning of migrant children found they were at an increased risk of non-fatal drowning, often in natural waterways, however they did not specifically separate out drowning location [8]. A third study exploring unintentional drowning mortality rates across 60 countries identified that natural waterways were high (e.g. 93% in Finland, 87% in Panama and 85% in Lithuania), again not identifying specific aquatic locations within the broad grouping of natural waterways [9]. These studies continue to highlight the challenges around understanding river-specific drowning [4].

Factors identified in the literature that have been found to increase the likelihood of fatal drowning in rivers include: being male, alcohol, swimming and the use of watercraft and a range of ages, with no consistency between publications [4]. A recent systematic literature review identified gaps in the published literature including the need for a consistent definition, national population level studies focusing solely on rivers, clarity on risk factors for river drowning and evidence for the effectiveness of proposed prevention strategies to reduce fatal river drowning [4].

To date there has been little epidemiological research into drowning deaths in rivers, due in large part to the difficulty in identifying the specific location of rivers within the coding (e.g. ICD) utilised for location of drowning in hospital and causes of death data [4]. Research has highlighted the shortcomings of the ICD coding framework which leads to underestimation of overall drowning [10–11]. A lack of specificity for location of drowning within the ICD framework sees location of drowning segmented into swimming pool, bathtub and natural waterway (a catch-all code that includes beach, ocean, river, lake etc) [12]. In Australia coronial data provides the most comprehensive and accurate opportunity to explore the casual factors of drowning deaths in detail [13].

Nationally, rivers have been identified as accounting for a fifth (20.3%) of all unintentional drowning deaths in Australia between 2002–2007 [14], making rivers the leading location for drowning in Australia. The prevention of unintentional river drowning deaths is of concern and has been identified by the Australian Water Safety Council (AWSC) as a key priority area for the drowning prevention sector in their bid to achieve a 50% reduction in drowning by the year 2020 [15]. There is a need for in-depth epidemiological analysis to identify risk factors to inform strategies for prevention. As such this paper aims to, for the first time, undertake a total population study from Australia describing unintentional fatal drowning in rivers.

## Methods

Data for the 10 year period 1 July 2002 to 30 June 2012 was sourced through privileged access to the Australian National Coronial Information System (NCIS) (JHREC—CF/15/13552). Ethical approval for the project was also provided by James Cook University (HREC—H6282). Cases in the NCIS remain open (i.e. under investigation) until such time as the Coroner makes a ruling as to cause of death and the case is closed. This specific 10 year period has been chosen to minimise the number of cases still under coronial investigation and therefore strengthen the data presented. At time of analysis, 93.1% of cases were closed. For open cases, data is correct as at 1 October, 2015.

Data relates to unintentional fatal drowning only. This is because different prevention strategies would need to be considered for intentional (i.e. self-harm, homicide, infanticide) injuries (including drowning) [16]. Deaths as a result of crocodile or shark attack were also excluded. Cases where intent was unlikely to be known or where the coroner has made an open finding were included. Cases were included where the primary medical cause of death was drowning, or drowning was a contributing factor. Cases where the victim was found in the river but the cause of death did not mention drowning (e.g. multiple drug toxicity or blunt force trauma) were not included.

Rivers, creeks and streams were defined as “…A natural waterway that may be fed from other rivers or bodies of water draining water away from a ‘catchment area’ to another location…” [4,17] and “…can vary in water flow, length, width and depth…” [18]. Non-aquatic transport relates to means of transport not primarily designed or intended for aquatic use such as motor vehicles, motorbikes, tractors, bicycles and aeroplanes among others. A pre-existing medical condition was defined as a disease or injury that was present prior to the drowning event that was documented in either the pathology/autopsy report or coronial finding. Categories of medical conditions were coded using the International Statistical Classification of Diseases and Related Health Problems 10th Revision [12].

Variables collected included age, sex, activity prior to drowning, time of day of drowning, day of the week and season of drowning, alcohol involvement, geographical location of incident, residential and incident postcode, remoteness classification of incident postcode, visitor status and Indigenous status [17]. Indigenous status refers to those known to be Aboriginal, Torres Strait Islander or both Aboriginal and Torres Strait Islander. Where Indigenous status was unknown, this was assumed to be ‘no’ for the purposes of analysis.

Due to difficulties around interpreting blood alcohol content (BAC) for drowning victims [19], alcohol involvement was deemed where a BAC was available (either in the autopsy or toxicology report) and was ≥0.05% (that is 0.05 grams of alcohol in every 100 millilitres of blood). Cases where alcohol was known to be consumed but no BAC was available were deemed unknown for alcohol involvement.

Time of incident was coded into four groupings for analysis: morning (6:01am to 12pm), afternoon (12:01pm to 6pm), evening (6:01pm to 12am) and early morning (12:01am to 6am). For the time of incident variable, where time could not be determined a coding of 9999 (unknown) was used.

The remoteness classification of an incident postcode is calculated based on a range of factors including distance and isolation from major services. The remoteness classification was coded to the Australian Standard Geographical Classifications (ASGC) and calculated using the Doctor Locator website, a site which provides ASGC classification by inputting a postcode [20]. Drowning rates per 100,000 population were calculated using population data from the Australian Bureau of Statistics (ABS) [21] and excluded international tourists. Drowning rates by remoteness of incident location (for Indigenous and non-Indigenous people) were calculated using Census population data by remoteness classification for the years 2001 [22], 2006 [23] and 2011 [24] and averaged out to determine average population and yearly drowning rate.

Visitor status was calculated by determining the distance, in kilometres, between the residential and incident postcodes using Google Maps [25]. A distance of 100km or less was classified as ‘Not A Visitor’; those who resided within the same State or Territory with a distance greater than 100km, were classified as an ‘Intrastate Visitor’; those who drowned in a different State or Territory and were greater than 100km from where they resided were classified as ‘Interstate Visitors’. Those with a residential postcode of 7777 (i.e. live overseas) were classified as ‘International Tourists’. Children were defined as aged 0–17 years and adults were defined as aged 18 years and over. In Australia, 18 years is the age a child reaches adulthood for the purposes of the criminal law [26].

Data coding and analysis was conducted in IBM SPSS V20 [27]. Descriptive statistics and chi squared analysis were utilised. A modified Bonferroni test suggested by Keppel [28] has been applied and therefore statistical significance was deemed p<0.03 (Table 1) and p<0.04 (Tables 2 and 3) respectively. Non-parametric chi squared analysis was also conducted using the proportional basis of the population as the assumed outcome numbers.

**Table 1:** Drowning deaths in rivers by sex, age group, state or territory of incident, Aboriginal and/or Torres Strait Islander status and remoteness classification of incident location, crude drowning rate per 100,000 population and relative risk (RR) with 95% confidence interval (CI), Australia, 2002/03 to 2011/12 (N = 770).

	Total	Crude rate / 100,000 population	Relative Risk (RR) with 95% Confidence Interval (CI) comparing drowning deaths in rivers to the relevant population	Χ^2^ (p value) ^a^
Total	770	0.36		
**Sex**				
Male	619	0.59	4.15 (3.47–4.95)	284.4 (p<0.001)
Female	151	0.14	1 ^b^	
**Age Group**				
0–4 years	32	0.23	1	56.6 (p<0.001)
5–9 years	26	0.19	0.82 (0.49–1.38)	
10–14 years	28	0.20	0.86 (0.52–1.43)	
15–17 years	27	0.32	1.37 (0.82–2.28)	
18–24 years	91	0.44	1.87 (1.25–2.79)	
25–34 years	110	0.37	1.57 (1.06–2.33)	
35–44 years	106	0.34	1.47 (0.99–2.19)	
45–54 years	105	0.36	1.55 (1.04–2.30)	
55–64 years	103	0.44	1.89 (1.27–2.81)	
65–74 years	64	0.43	1.82 (1.19–2.78)	
75+ years	78	0.60	2.55 (1.69–3.85)	
**State or Territory of Incident Location**				
Australian Capital Territory (ACT)	5	0.14	1	260.6 (p<0.001)
New South Wales (NSW)	268	0.39	2.69 (1.11–6.52)	
Northern Territory (NT)	43	1.98	13.73 (5.44–34.66)	
Queensland (QLD)	217	0.52	3.62 (1.49–8.79)	
South Australia (SA)	34	0.21	1.49 (0.58–3.81)	
Tasmania (TAS)	35	0.70	4.90 (1.92–12.50)	
Victoria (VIC)	107	0.20	1.42 (0.58–3.49)	
Western Australia (WA)	61	0.28	1.96 (0.79–4.88)	
**Aboriginal and/or Torres Strait Islander Status ^c^**				
Yes	82	1.32	3.93 (3.12–4.94)	164.2 (p<0.001)
No	688	0.34	1	
**Remoteness Classification of Incident Location ^d^**				
Major Cities	217	0.16	1	1333.4 (p<0.001)
Inner Regional	226	0.58	3.62 (2.58–5.09)	
Outer Regional	196	1.01	6.37 (4.47–9.06)	
Remote	49	1.58	9.95 (5.65–17.53)	
Very Remote	82	4.57	28.76 (18.08–45.73)	

^a^ A modified Bonferroni test has been applied meaning statistical significance is deemed at p<0.03.

^b^ Where relative risk (RR) was calculated, the group with the lowest rate was used as the reference point.

^c^ Where Indigenous status was unknown, this was assumed to be ‘no’ for the purposes of analysis.

^d^ Population data is only available for three population census years (2001, 2006 and 2011), therefore a 12 year average for deaths and a three year average for population have been used to calculate crude rates.

**Table 2:** River drowning deaths by mean age, sex and children or adults by activity immediately prior to drowning, presence of alcohol, pre-existing medical condition and Aboriginal and/or Torres Strait Islander status, and relative risk (RR) with 95% confidence interval (CI), Australia, 2002/03 to 2011/12 (N = 770).

	Total		Mean Age with 95% Confidence Interval (CI)	Male		Female ^a^		Relative Risk (RR) with 95% Confidence Interval (CI) comparing females to males for river drowning deaths	Χ^2^ (p value) ^c^	Children ^a^		Adults		Relative Risk (RR) with 95% Confidence Interval (CI) comparing children to adults for river drowning deaths	Χ^2^ (p value) ^c^
	N	%		N	%	N	%			N	%	N	%		
	770	100	42.0 (40.4–43.6)	619	80.4	151	19.6			113	14.7	657	85.3		
**Activity Immediately Prior to Drowning**															
Falls	164	21.3	44.5 (40.1–48.8)	123	75	41	25	0.99 (0.72–1.36)	3.3 (p = 0.069)	38	23.2	126	76.8	0.57 (0.42–0.77)	6.3 (p = 0.012)
Non-aquatic Transport	140	18.2	44.8 (40.9–48.6)	92	65.7	48	34.3	0.47 (0.35–0.63)	22.4 (p<0.001)	24	17.1	116	82.9	0.83 (0.56–1.23)	0.0 (p = 0.916)
Swimming	125	16.2	31.6 (28.9–34.3)	109	87.2	16	12.8	1.66 (1.01–2.72)	5.1 (p = 0.023)	23	18.4	102	81.6	0.76 (0.51–1.14)	0.3 (p = 0.606)
Watercraft	107	13.9	41.4 (37.7–45.1)	98	91.6	9	8.4	2.66 (1.37–5.13)	10.9 (p = 0.001)	11	10.3	96	89.7	1.50 (0.83–2.71)	4.0 (p = 0.047)
Jumped In	37	4.8	26.3 (22.1–30.5)	35	94.6	NP ^b^	NP	4.27 (1.04–17.55)	5.3 (p = 0.022)	9	24.3	28	75.7	0.54 (0.26–1.10)	1.6 (p = 0.211)
Swept Away	23	3	48.3 (38.3–58.3)	11	47.8	12	52.2	0.22 (0.10–0.50)	15.3 (p<0.001)	NP	NP	20	87	1.15 (0.35–3.80)	0.3 (p = 0.620)
Other	51	6.6	43.6 (38.5–48.8)	49	96.1	NP	NP	5.98 (1.47–24.30)	9.0 (p = 0.003)	NP	NP	50	98	8.60 (1.20–61.63)	8.8 (p = 0.003)
Unknown	123	16	49.8 (46.4–53.2)	102	82.9	21	17.1			NP	NP	119	96.7		
**Presence of Alcohol (≥0.05)**															
Yes	196	25.5	41.7 (39.4–44.0)	166	84.7	30	15.3	1.35 (0.96–1.91)	2.7 (p = 0.098)	7	3.6	189	96.4	4.64 (2.24–9.61)	33.4 (p<0.001)
No	321	41.7	41.3 (38.5–44.0)	253	78.8	68	21.2			72	22.4	249	77.6		
Unknown	253	32.9	43.3 (40.5–46.1)	200	79.1	53	20.9			34	13.4	219	86.6		
**Pre-existing Medical Condition**															
Yes	288	37.4	53.5 (51.1–55.8)	234	37.8	54	35.8	1.06 (0.83–1.34)	0.2 (p = 0.668)	14	4.9	274	95.1	3.37 (2.04–5.54)	72.6 (p<0.001)
No	211	27.4	26.8 (24.3–29.3)	169	27.3	43	28.5			72	34.1	139	65.9		
Unknown	271	35.2	41.8 (39.4–44.3)	216	34.9	54	35.8			27	10	244	90		
**Aboriginal and/or Torres Strait Islander Status**															
Yes	82	10.6	31.5 (28.0–35.0)	66	80.5	16	19.5	1.01 (CI: 0.60–1.69)	0.0 (p = 0.912)	20	24.4	62	75.6	0.53 (0.34–0.85)	5.0 (p = 0.025)
No	584	75.8	43.2 (41.3–45.0)	467	80	117	20			86	14.7	498	85.3		
Unknown	104	13.5	44.1 (40.1–48.0)	86	82.7	18	17.3			7	6.7	97	93.3		

^a^ Where relative risk (RR) was calculated, females and children have been used as the control groups

^b^ NP = not presented for variables where case numbers amounted to four deaths and under

^c^ A modified Bonferroni test has been applied meaning statistical significance is deemed at p<0.04.

**Table 3:** River drowning deaths by sex and children or adults by visitor status, remoteness classification of incident, season of drowning incident and time of day of drowning incident and relative risk (RR) and 95% confidence interval (CI), Australia, 2002/03 to 2011/12 (N = 770).

	Total		Male		Female ^a^		Relative Risk (RR) with 95% Confidence Interval (CI) comparing females to males for river drowning deaths	Χ^2^ (p value) ^d^	Children ^a^		Adults		Relative Risk (RR) with 95% Confidence Interval (CI) comparing children to adults for river drowning deaths	Χ^2^ (p value) ^d^
	N	%	N	%	N	%			N	%	N	%		
	770	100.0	619	80.4	151	19.6			113	14.7	657	85.3		
**Visitor Status**														
Not a Visitor	570	74.0	454	79.6	116	20.4	0.95 (0.86–1.05)	0.4 (p = 0.508)	87	15.3	483	84.7	0.95 (0.85–1.07)	0.1 (p = 0.711)
Visitor–Intrastate	110	14.3	87	79.1	23	20.9	0.92 (0.60–1.41)	0.1 (p = 0.755)	21	19.1	89	80.9	0.73 (0.47–1.12)	1.7 (p = 0.192)
Visitor—Interstate	46	6.0	40	87.0	6	13.0	1.63 (0.70–3.76)	1.4 (p = 0.234)	NP ^b^	NP	42	91.3	1.81 (0.66–4.94)	1.5 (p = 0.217)
Visitor—Overseas	21	2.7	18	85.7	NP	NP	1.46 (0.44–4.90)	0.4 (p = 0.519)	0	0.0	21	100.0	UTBC ^c^	3.8 (p = 0.051)
Unknown	23	3.0	20	87.0	NP	NP			NP	NP	22	95.7		
**Remoteness Classification of Incident Location**														
Major Cities	217	28.2	183	84.3	34	15.7	1.31 (0.95–1.81)	3.0 (p = 0.084)	26	12.0	191	88.0	1.26 (0.88–1.81)	1.8 (p = 0.186)
Inner Regional	226	29.4	171	75.7	55	24.3	0.76 (0.59–0.97)	4.5 (p = 0.033)	33	14.6	193	85.4	1.01 (0.74–1.37)	0.0 (p = 0.970)
Outer Regional	196	25.5	161	82.1	35	17.9	1.12 (0.82–1.54)	0.5 (p = 0.474)	29	14.8	167	85.2	0.99 (0.70–1.39)	0.0 (p = 0.956)
Remote	49	6.4	39	79.6	10	20.4	0.95 (0.49–1.86)	0.0 (p = 0.884)	7	14.3	42	85.7	1.03 (0.48–2.24)	0.0 (p = 0.937)
Very Remote	82	10.6	65	79.3	17	20.7	0.93 (0.56–1.54)	0.1 (p = 0.787)	18	22.0	64	78.0	0.61 (0.38–0.99)	3.9 (p = 0.049)
**Season of Drowning Incident**														
Summer	295	38.3	239	81.0	56	19.0	1.04 (0.83–1.31)	0.1 (p = 0.730)	53	18.0	242	82.0	0.79 (0.63–0.98)	4.1 (p = 0.042)
Autumn	145	18.8	112	77.2	33	22.8	0.83 (0.59–1.17)	1.1 (p = 0.289)	18	12.4	127	87.6	1.21 (0.77–1.91)	0.7 (p = 0.393)
Winter	157	20.4	128	81.5	29	18.5	1.08 (0.75–1.55)	0.2 (p = 0.687)	16	10.2	141	89.8	1.52 (0.94–2.44)	3.1 (p = 0.075)
Spring	173	22.5	140	80.9	33	19.1	1.03 (0.74–1.45)	0.0 (p = 0.840)	26	15.0	147	85.0	0.97 (0.67–1.40)	0.0 (p = 0.881)
**Time of Day of Drowning Incident**														
Morning	147	19.1	123	83.7	24	16.3	1.25 (0.84–1.86)	1.1 (p = 0.306)	23	15.6	124	84.4	0.93 (0.62–1.38)	0.0 (p = 0.944)
Afternoon	321	41.7	254	79.1	67	20.9	0.92 (0.76–1.13)	1.0 (p = 0.331)	65	20.2	256	79.8	0.68 (0.56–0.82)	10.3 (p = 0.001)
Evening	160	20.8	128	80.0	32	20.0	0.98 (0.69–1.38)	0.1 (p = 0.796)	16	10.0	144	90.0	1.55 (0.96–2.49)	4.7 (p = 0.030)
Early Morning	76	9.9	63	82.9	13	17.1	1.18 (0.67–2.09)	0.3 (p = 0.571)	5	4.4	72	94.7	2.48 (1.02–6.00)	5.3 (p = 0.021)
Unknown	66	8.6	51	77.3	15	22.7			5	7.6	61	92.4		

^a^ Females and children have been used as the control groups

^b^ NP = not presented for variables where case numbers amounted to four deaths and under

^c^ UTBC = unable to be calculated

^d^ A modified Bonferroni test has been applied meaning statistical significance is deemed at p<0.04.

Relative risk (RR) for risk factors within river drowning victims was calculated, along with a 95% Confidence Interval (CI). When calculating relative risk, females and children were used as the control groups. Relative risk and chi squared analysis were conducted without the ‘unknown’ variable–e.g. presence of alcohol was calculated using the ‘yes’ and ‘no’ variables only. Where variable analysis presents cases of four or less, the term NP (not presented) has been used for both numbers and percentages in Tables 1 and 2.

## Results

During the study period 2,892 people died as a result of unintentional fatal drowning. Of these, 770 people drowned in rivers (26.6%), 487 in Ocean / Harbour locations (16.8%) and 440 at Beaches (15.2%), making rivers the leading location for drowning in Australia.

**Fig 1:**
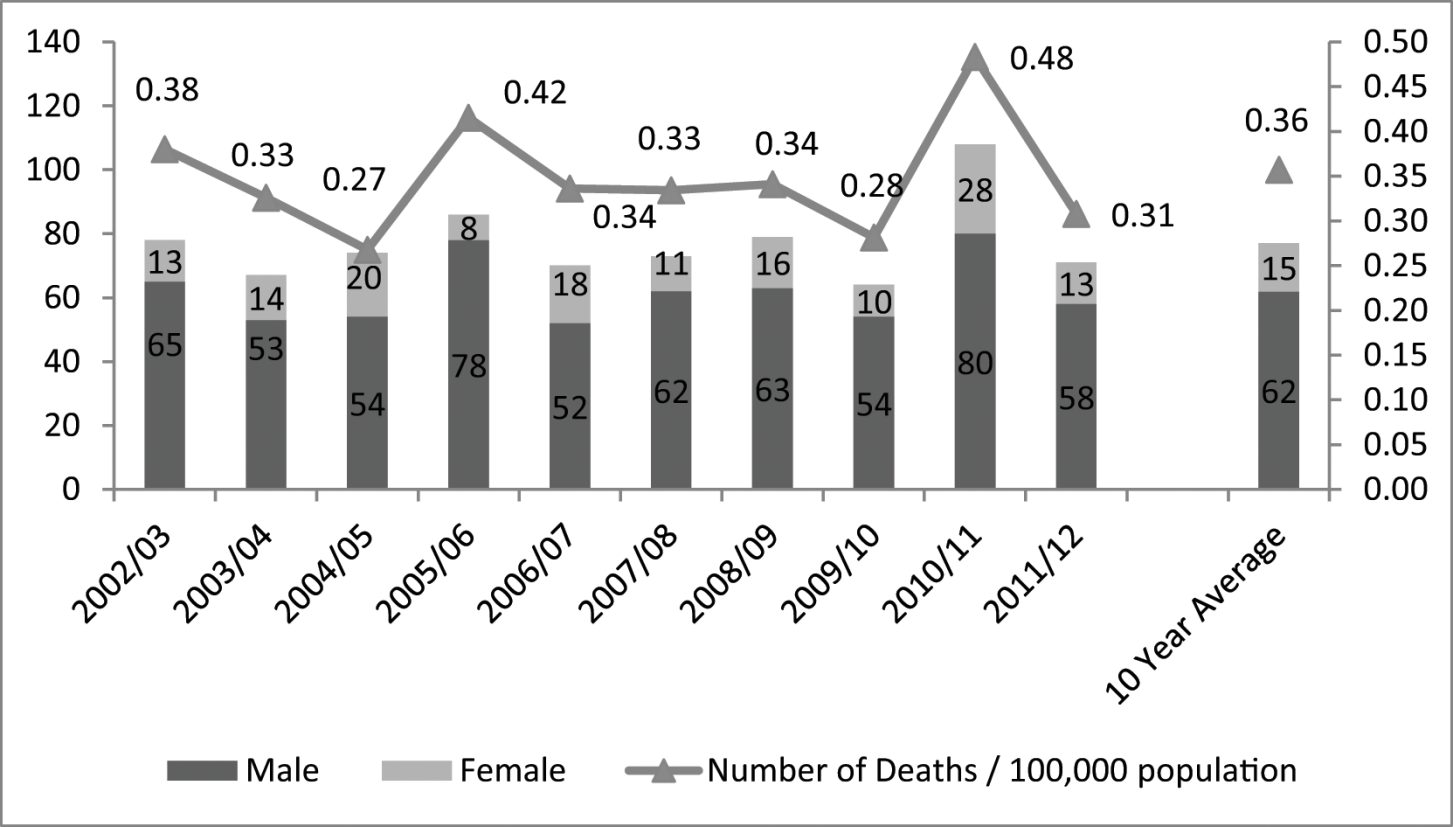
Drowning deaths in rivers by financial year, number by sex and crude rate per 100,000 population, Australia, 2002/03 to 2011/12 (N = 770).

Annual crude drowning rates per 100,000 population have remained steady across the study period ranging from a high of 0.48 in 2010/11 (the year of the Queensland floods which claimed the lives of 22 people [29]) to a low of 0.27 in 2004/05. (Fig 1)

Males accounted for 80.4% (a rate of 0.59 per 100,000 population) of drowning fatalities in rivers. When compared to females drowning in rivers, males drown in rivers at a rate that is 4.15 times higher (X^2^ = 284.4; p<0.001) (Table 1).

**Fig 2:**
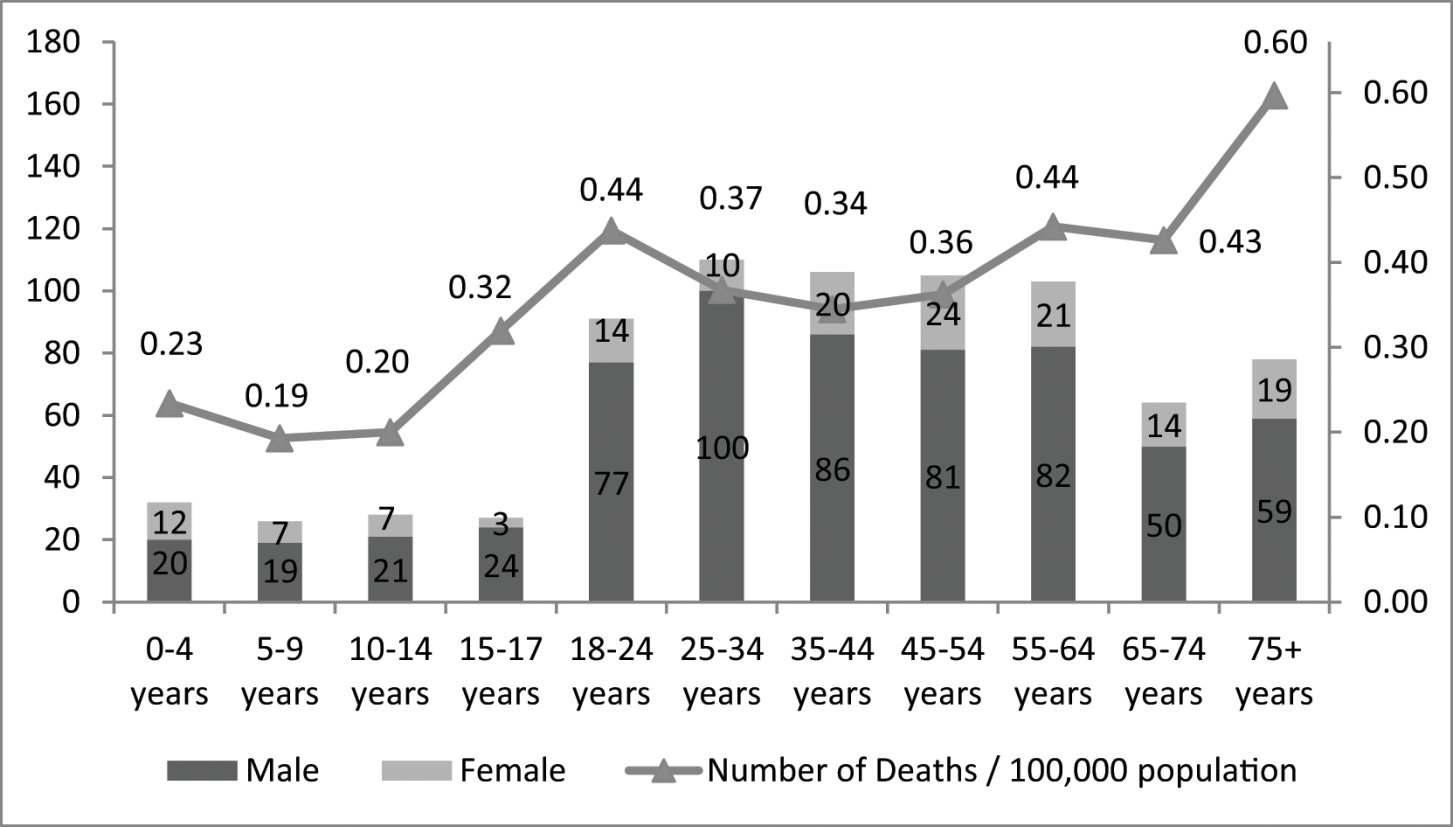
Drowning deaths in rivers by age group and sex, and crude rate by age group per 100,000 population, Australia, 2002/03 to 2011/12 (N = 770).

The mean age of river drowning victims was 42.0 years (males 41.7 years; females 43.5 years) (Table 2). Over half (55.1%) of all deaths occur between 25–64 years. People aged 75+ years recorded the highest age-specific drowning rate per 100,000 population in rivers at 0.60, with the lowest rate seen in the 5–9 years age group (0.19). (Fig 2)

People aged 75 years and over drown in rivers at a rate that is 2.55 (CI: 1.69–3.85) times that of children aged 0–4 years. This was followed by 55–64 year olds at 1.89 times (CI: 1.27–2.81) and 18–24 year olds at 1.87 times (CI: 1.25–2.79). Age was found to be statistically significant for drowning in rivers (X^2^ = 56.6; p<0.001). (Table 1)

Aboriginal and Torres Strait Islanders drown in rivers at a rate that is 4.62 (CI: 3.67–5.83) times that of non-Indigenous people in rivers (Table 1). Indigenous river drowning victims also recorded a younger mean age (31.5 years) when compared to non-Indigenous victims (43.2 years) (Table 2).

The prevalence of male drowning was most pronounced in the 25–34 years age group (90.9%) male followed by the 15–17 years age group (88.9% male). (Fig 2)

A fall into water was the leading activity prior to drowning in rivers (21.3%), followed by incidents involving non-aquatic transport (18.2%) and swimming (16.2%). Activity immediately prior to drowning was unknown in 16.0% of cases. (Table 2)

When examining activity prior to drowning in rivers by sex of the victim, females were 2.27 and 4.45 times more likely respectively to drown as a result of incidents involving non-aquatic transport (χ^2^ = 22.4; p<0.04) and being swept away by floodwaters (χ^2^ = 15.3; p<0.04). When compared to females, males were 2.66 more likely to drown in rivers as a result of watercraft incidents (χ^2^ = 10.9; p<0.04) and 4.27 times more likely as a result of jumping in (χ^2^ = 5.3; p<0.04). (Table 2)

When comparing activity prior to drowning in rivers between children and adults, children were 1.75 times more likely to drown as a result of a fall into water (χ^2^ = 6.3; p<0.04) and adults were 8.60 times more likely to drown in rivers when participating in an activity coded as other when compared to children (χ^2^ = 8.8; p = 0.04). (Table 2)

Aboriginal and Torres Strait Islanders accounted for 10.6% of all drowning deaths in rivers, of these 24.4% were children and 75.6% adults. (Table 2) Almost three quarters (74.0%) of all river drowning victims were local to the area where they drowned, a further 20.3% were intrastate or interstate travellers. Just 2.7% of all river drowning victims were international tourists (of which all were adults). (Table 3)

The largest proportion of river drowning deaths occurred in inner regional locations (29.4%). Proportionally, compared to population distribution, very remote areas recorded the highest crude rate of fatal drowning with an average rate of fatal drowning at 4.57 per 100,000 population, with people in very remote areas drowning in rivers at a rate of 28.76 (CI: 18.08–45.73) times that of people who drown in rivers in major cities… Over half (χ^2^ = 107.3; p<0.05) of Indigenous victims drowned in rivers in very remote areas. (Table 3) The crude rate of drowning by remoteness classification for Indigenous river drowning victims was higher across all remoteness classifications when compared to non-Indigenous river drowning victims, with the exception of remote areas. Indigenous people drowned at a rate of 0.33 in major cities, compared to 0.16 for non-indigenous people, 1.29 compared to 0.56 for inner regional, 2.12 compared to 0.95 for outer regional, 1.31 compared to 1.62 for remote and 5.12 compared to 4.20 for very remote areas. Over a third of all river drowning victims (37.4%) were known to have a pre-existing medical condition (95.1% adults) (Table 2). The most common categories of medical conditions were diseases of the circulatory system (such as ischemic heart disease, hypertension and cardiomyopathy) (14.8% of all drowning victims in rivers), followed by mental and behavioural disorders (such as dementia, autism and depression) (11.4%) and diseases of the nervous system (such as epilepsy and multiple sclerosis) (4.4%).

Almost half of all river drowning cases (41.7%) occurred in the afternoon. (Table 2) The time of day of the drowning incident was not found to be significant for adults, however children were more likely (χ^2^ = 10.3; p<0.04) to drown in rivers in the afternoon (Table 3)

Alcohol was known to be present (BAC readings of ≥0.05%) in 25.5% of cases (6.2% children; 28.8% adults), however information on BAC was missing in 32.9% of cases. The mean age of river drowning victims who recorded positive readings for alcohol was 41.7 years (male 40.7 years; female 46.9 years). The mean BAC recorded was 0.21% (male 0.21%; female 0.20%). (Table 2)

## Discussion

Preventing drowning in rivers is a significant challenge due to the number and locations of rivers, diversity of activity prior to drowning and a lack of known prevention strategies [4]. Seven areas have been identified where further work in developing prevention strategies might help in reducing river drowning deaths, noting that further work is required to understand exposure as this influences potential risk.

### Identifying river drowning

Drowning in rivers is a little explored area, compounded by the lack of a specific location code within the ICD coding framework which is used internationally to categorise cause of death [4]. This study, for the first time, describes the epidemiology of river drowning deaths at a country level as its sole focus. There is a need for a greater understanding of river drowning in Australia given the burden, and we hypothesize that rivers may represent similar burdens internationally [9]. Further research on river drowning deaths is needed as rivers have a different morphology from other aquatic locations [30]. Alternative coding mechanisms need to be employed [31] to better identify rivers as a location for drowning and develop more effective prevention strategies.

Further research is also required around exposure. Exposure is one of the significant challenges for understanding risk around drowning in general [4] (and river drowning specifically) and is a challenge to collect at the macro level (i.e. how often they visit a river location in a given time period) but especially at the micro level (i.e. what was the person doing at the location and for how long), ignoring the challenges around water depth, speed and temperature among other factors.

To our knowledge two Australian studies have explored river exposure. Using a population health survey, the first study compared drowning mortality and hospitalised morbidity rates by population rates of exposure by location and activity [32]. It should be noted that this study only examined people in one Australian state (New South Wales) and grouped rivers into broader exposure to water (e.g. swimming pools, beach, lake, river, creek, stream or dam). The second study, which also utilised a community survey, only looked at the activity of swimming in a river (albeit nationally), which found that the majority of people (80%) had not swum in a river over the previous 12 months [33]. Thus rates based on exposure are likely to be higher than what is reported for the whole population in this paper.

### Role of alcohol in river drowning

Alcohol is known to be a risk factor for drowning [13]. This study has found high blood alcohol levels in river drowning victims, an average BAC of 0.20%, four times the legal driving limit in Australia. Such significant levels of alcohol are likely to increase one’s risk of drowning. Proposed strategies such as greater enforcement [34] (more active breathalysing of skippers, the establishment of no drinking zones around popular river locations and associated penalties) as well as increased public education and awareness of the role of alcohol in river drowning should be explored. Any preventative strategies employed must be evaluated to determine effectiveness [4].

### Pre-existing medical conditions

Two in five adult river drowning victims were known to have a pre-existing medical condition, however it is unknown if this contributed to their death. Further work is required to compare the prevalence of particular pre-existing medical conditions within the river drowning cohort and the Australian population to determine risk. A medical check-up, particularly for those who go diving [35–37], has been recommended and, as such, should be tested to see if it may be a viable strategy for the prevention of drowning in rivers.

### Males and females

Males made up the majority of river drowning deaths, however the profiles differed. Females were proportionately more likely to drown as a result of incidents involving non-aquatic transport (2.27 times) and being swept away by floodwaters (4.45 times) and males as a result of watercraft incidents (2.66 times) and jumping in (4.27 times). Males have been identified as greater risk takers, including for drowning [38] and lessons from other areas of injury prevention [39–40] may be transferable to the prevention of drowning deaths. This is an area that needs to be explored in greater detail.

There is a need for better understanding of river usage by gender (exposure studies) to inform prevention. Further work is also needed to understand the nature of watercraft drowning incidents in rivers. For example a study by Smith identified the need to targeting alcohol and boating safety campaigns to both skippers and passengers and to broaden the prohibition of drinking to not only being while the boat is under way [34].

### Children and adults

Adults and children have different profiles when it comes to drowning in rivers. Children were more likely to drown as a result of falls (unintended access) (33.6%) and to drown in the afternoon (57.5%), whereas, when compared to children, adults were at an increased risk of drowning in rivers if alcohol was known to be involved (28.8%).

Supervision has been recommended as a strategy for the prevention of drowning in children [41–43], however there is little evidence about this being an effective strategy for river drowning, nor information around how this strategy should be communicated [44–45]. Lifejackets have also been proposed as another prevention strategy [46–47]. However all strategies need to be validated for effectiveness at a river setting.

### Visitor status of river drowning victims

While the media [35, 48] may more commonly highlight the drowning of a tourist, the majority (74.0%) of river drowning victims are locals. Prevention strategies targeted at local people are likely to have the greatest impact, however this is a challenge in Australia based on size and population distribution. Other challenges exist in communicating safety information as there may be a familiarity or perceived ‘local knowledge’ that may lead to people underestimating their drowning risk. This should be explored further through exposure and attitudinal studies.

### Remoteness of river drowning incident location

As river drowning locations became more remote, the relative risk based on the population residing in those areas increased, with very remote locations recording fatal unintentional drownings in rivers at a rate (4.57) that is 28.76 times higher than river drowning deaths in major cities (0.16). Almost half (45.1%) of all Indigenous victims drowned in rivers in very remote areas, recording a rate of 5.12 drowning deaths per 100,0000 population compared to 4.20 for non-Indigenous people within the same remoteness classification. Strategies for the prevention of drowning in isolated areas will be a major challenge due to the dispersion of the population over large areas as well as the difficulties and costs in reaching large numbers of people efficiently [49–51]. Indigenous-specific river drowning prevention strategies must be developed in partnership with Indigenous communities [52].

It has been argued that all adults [53–54] should be equipped with cardio-pulmonary resuscitation (CPR) skills. There is a lack of information on CPR with respect to river drowning victims. Therefore it was not included and further research is required into the use of CPR and its effectiveness in the prevention of river drowning deaths. Such skills may improve bystanders’ response in locations where timely medical assistance may be difficult due to remoteness. Further research is required to determine if CPR was performed in these cases, if the victim was alone when they drowned, whether emergency services were called, and if any of these factors would have varied the fatal outcome.

Any strategies which are delivered need to include an evaluation component, as there is a lack [4] of evidence to show what works and does not work to prevent drowning in rivers. Understanding drowning in rivers may also help prevent drowning internationally, as rivers are a common location for drowning across the world [1, 4].

### Limitations

- Cases where intent was unlikely to be known or where the coroner has made an open finding were included. This may overestimate the number of unintentional fatal drownings in rivers as 10.1% of closed cases recorded an intention of ‘unlikely to be known’ and a further 2.5% of cases were coded ‘undetermined intent’ as cases were under investigation within the NCIS. However this information is also cross checked with media and other reports so the numbers should not change significantly.
- Information for some variables may be limited due to 6.9% of cases being open (i.e. under investigation) within the coronial system at the time of publishing. This limits the access of the researcher to case files such as coronial finding and autopsy, toxicology and police reports. This affects variables such as pre-existing medical condition, activity prior to drowning and presence of alcohol.
- Access to toxicology and autopsy reports is not available in all cases (e.g. case files not electronically uploaded or autopsy or toxicology testing is not performed, or the case is under investigation), thus impacting our understanding of the involvement of alcohol and drugs. Data on the victim's BAC level was unknown in 32.9% of cases.
- Calculations for crude fatal drowning rates by population of remoteness classifications uses an average from three years 2001, 2006 and 2011 (Australian Census years) for population and drowning data from 2002/03 to 2011/12. This may produce rates that are not as accurate as if population data was available for each year of drowning data.
- There is limited exposure data available around drowning in general and river drowning specifically. Therefore, calculations of relative risk include only people in the study (i.e. those people who died as a result of an unintentional drowning in a river) and do not take into account the total exposed population.
- Cadavers that have been submerged in water for a period of time naturally produce alcohol [19]. Therefore due to a lack of reliable information on the amount of time between death and autopsy/toxicological testing, BACs represented in this dataset may be artificially inflated due to decomposition.
- Where Indigenous status was unknown, for the purposes of calculating rates, the ‘unknown’ group were included with the ‘no’. This may underestimate the number of Indigenous people within the population. However we note that the average age of the ‘unknown’ group was similar to that of the ‘no’ group (Table 2).

## Conclusion

Rivers were the leading location for unintentional fatal drowning in Australia between 2002 and 2012. This study has highlighted the differences in river drowning deaths between males and females, adults and children. Locals, adults who consume alcohol, those who fall in or use watercraft, and those in very remote locations were common groupings among drowning deaths in rivers in Australia. There is a need for the development, implementation and evaluation of strategies to prevent further drowning in Australian rivers.

## References

[1] World Health Organisation. Global Report on Drowning: Preventing a Leading Killer Geneva: World Health Organisation, 2014.

[2] Rahman A, Giashuddin SM, Svanstrom L, Rahman F. Drowning–A major but neglected child health problem in rural Bangladesh: Implications for low income countries. International Journal of Injury Control and Safety Promotion. 2006; 13(2):101–105. 16707346 10.1080/17457300500172941

[3] Thompson DC, Rivara FP. Pool fencing for preventing drowning in children (Cochrane Review) [Web Page]. The Cochrane Library; 2001 [updated 19 September 1997; cited 2003 9-01-2003]. Issue 2:10.1002/14651858.CD001047 PMC8407364 10796742

[4] Peden AE, Franklin RC, Leggat PA. Fatal river drowning: the identification of research gaps through a systematic literature review. Inj Prev 2016; 22:202–209 10.1136/injuryprev-2015-041750 26728005 PMC4893118

[5] Bessereau J, Fournier N, Mokhtari T, Brun P, Desplantes A, Grassineau D, et al Epidemiology of unintentional drowning in a metropolis of the French Mediterranean coast: a retrospective analysis (2000–2011). International Journal of Injury Control and Safety Promotion. 2015 10.1080/17457300.2015.1047862 26082429

[6] Zhu Y, Jiang X, Li F, Chen J. Mortality among drowning rescuers in China, 2013: a review of 225 rescue incidents from the press. BMC Public Health. 2015 10.1186/s12889-015-2010-0 PMC4496822 26156246

[7] Prameprart M, Lim A, Tongkumchum P. Modelling Unintentional Drowning Mortality Rates in Thailand, 200–2009. Asia-Pacific Journal of Public Health. 2015; 27(2); NP2471-NP2479. 10.1177/1010539513488796 23761591

[8] Zhu Y, Xu G, Li H, Huang Y, Ding K, Chen J. Epidemiology and risk factors for nonfatal drowning in the migrant children. Southeast Asian J Trop Med Public Health. 2015; 46(6); 1112–1123. 26867370

[9] Lin C, Wang Y, Lu T, Kawach I. Unintentional drowning mortality, by age and body of water: an analysis of 60 countries. Inj Prev. 2015; 21; e43–e50. 10.1136/injuryprev-2013-041110 24944343 PMC4501178

[10] Lu T, Lunetta P, Walker S. Quality of cause-of-death reporting using ICD-10 drowning codes: a descriptive study of 69 countries. BMC Medical Research Methodology. 2010; 10(1): 30. 20374660 10.1186/1471-2288-10-30 PMC2858216

[11] Lunetta P, Penttilä A, Sajantila A. Drowning in Finland: "external cause" and "injury" codes. Injury Prevention. 2002; 8(4): 342–344. 12460977 10.1136/ip.8.4.342 PMC1756588

[12] National Centre for Classification in Health Australia (2004). ICD-10-AM Tabular List of Diseases. Sydney.

[13] Driscoll T, Harrison JA, Steenkamp M. Review of the role of alcohol in drowning associated with recreational aquatic activity. Injury Prevention. 2004; 10: 107–113. 15066977 10.1136/ip.2003.004390 PMC1730083

[14] Franklin RC, Scarr JP, Pearn JH. Reducing drowning deaths: the continued challenge of immersion fatalities in Australia. Medical Journal of Australia. 2010; 192(3):123–126. 20121677 10.5694/j.1326-5377.2010.tb03448.x

[15] Australian Water Safety Council. Australian Water Safety Strategy 2012–15 Australian Water Safety Council, 2012.

[16] Mock C, Quansah R, Krishnan R, Arreola-Risa C, Rivara, F. Strengthening the prevention and care of injuries worldwide. The Lancet. 2004; 363 (9427): 2172–2179. 10.1016/S0140-6736(04)16510-0 15220042

[17] Royal Life Saving Society—Australia; 2014. Royal Life Saving Society—Australia Drowning Database Definitions and Coding Manual 2014.

[18] Australian Water Safety Council. A Guide to Water Safety Essentials for Local Governments. Sydney: 2008.

[19] Kugelberg FC, Jones AW. Interpreting results of ethanol analysis in postmortem specimens: A review of the literature. Forensic Science International. 2007; 165(1):10–29. 16782292 10.1016/j.forsciint.2006.05.004

[20] Australian Government Department of Health. Doctor Locator 2010 [26 June 2015]. Available: http://www.doctorconnect.gov.au/internet/otd/Publishing.nsf/Content/locator.

[21] Australian Bureau of Statistics. 3101.0—Australian Demographic Statistics, Jun 2014. 2014.Canberra: Australian Bureau of Statistics; Available: http://www.abs.gov.au/AUSSTATS/abs@.nsf/DetailsPage/3101.0Sep%202015?OpenDocument

[22] Australian Bureau of Statistics. 2001 Census QuickStats. 2006. Available: http://www.censusdata.abs.gov.au/census_services/getproduct/census/2001/quickstat/0?opendocument&navpos=220.

[23] Australian Bureau of Statistics. 2006 Census QuickStats. 2007. Available: http://www.censusdata.abs.gov.au/census_services/getproduct/census/2006/quickstat/0?opendocument&navpos=220.

[24] Australian Bureau of Statistics. 2011 Census QuickStats. 2013. Available: http://www.censusdata.abs.gov.au/census_services/getproduct/census/2011/quickstat/0?opendocument&navpos=220.

[25] Google. Google Maps (www.google.com.au/maps) 2015. Available: http://www.google.com.au/maps.

[26] Australian Government—Australian Law Reform Commission. Seen and heard: priority for children in the legal process (ALRC Report 84): Australian Government 2015. Available: http://www.alrc.gov.au/publications/18-childrens-involvement-criminal-justice-processes/age-thresholds-criminal-justice-pro. Accessed 7 August 2015

[27] SPSS Inc. IBM SPSS Statistics 20.0. Chicago, Illinois: IBM; 2010.

[28] Keppel G. (1991). Design and analysis: A researcher's handbook (3rd ed.). Englewood Cliffs, NJ: Prentice Hall.

[29] Queensland Floods Commission of Inquiry (2012). Queensland Floods Commission of Inquiry—Final Report March 2012. Queensland Floods Commission of Inquiry

[30] Vinet F, Lumbroso D, Defossez S, Boissier L. A comparative analysis of the loss of life during two recent floods in France: the sea surge caused by the storm Xynthia and the flash flood in Var. Natural Hazards. 2012; 61(3): 1179–1201.

[31] ICECI Coordination and Maintenance Group (2004). International Classification of External Causes of Injuries (ICECI) version 1.2.

[32] Mitchell RJ, Williamson AM, Olivier J. "Estimates of drowning morbidity and mortality adjusted for exposure to risk." Injury Prevention. 2010; 16(4): 261–266. 10.1136/ip.2009.024307 20696716

[33] Franklin RC. (2006). Evaluation of Bronze. Royal Life Saving Society Australia, Sydney.

[34] Smith GS, Keyl PM, Hadley JA, Bartley CL, Foss RD, Tolbert WG, et al "Drinking and Recreational Boating Fatalities: A Population-Based Case-Control Study." JAMA: The Journal of the American Medical Association. 2001; 286(23): 2974–2980. 11743838

[35] Peden AE, Franklin RC, Leggat PA. International Travelers and Unintentional Fatal Drowning in Australia—A 10 Year Review 2002–2012. Journal of Travel Medicine. 2016; 1–7: 10.1093/jtm/tav031 26883927

[36] Lippmann J, Pearn J. Snorkelling-related deaths in Australia, 1994–2006. Medical Journal of Australia. 2012; 197(4):230–232. 22900874 10.5694/mja11.10988

[37] Nakayama H, Smerz RW. Descriptive epidemiological analysis of diving accidents in Hawaii from 1983 to 2001. Hawaii Medical Journal. 2003 62(8): 165–70. 14533348

[38] Howland J, Hingson R, Mangione T, Bell N, Bak S. Why are most drowning victims men? Sex differences in aquatic skills and behaviours. American Journal of Public Health. 1996; 86(1): 93–96. 10.2105/AJPH.86.1.93 8561253 PMC1380371

[39] Turner C, McClure R. Age and gender differences in risk-taking behaviour as an explanation for high incidence of motor vehicle crashes as a driver in young males. International Journal of Injury Control and Safety Promotion. 2003; 10(3): 123–130. 10.1076/icsp.10.3.123.14560 12861910

[40] Wilson M, Daly M. Competitiveness, risk taking and violence: the young male syndrome. Ethology and Sociobiology. 1985; 6(1): 59–73. 10.1016/0162-3095(85)90041-X

[41] Kemp A, Sibert JR. Drowning and near drowning in children in the United Kingdom: lessons for prevention. BMJ 1992; 304: 1143–6. 1392791 10.1136/bmj.304.6835.1143 PMC1882129

[42] Pearn J, Nixon J. Prevention of childhood drowning accidents. The Medical Journal of Australia. 1977; 1: 616–618. 875811 10.5694/j.1326-5377.1977.tb130960.x

[43] Brenner R. and Committee on Injury Violence and Poison Prevention. Prevention of Drowning in Infants, Children, and Adolescents: Technical Report. Pediatrics. 2003; 112(2): 440–445. 12897306 10.1542/peds.112.2.440

[44] Bugeja L, Franklin RC. An analysis of stratagems to reduce drowning deaths of young children in private swimming pools and spas in Victoria, Australia. International Journal of Injury Control and Safety Promotion. 2012: 1–13. 10.1080/17457300.2012.717086 22950370

[45] Morrongiello BA, Schell SL. Child Injury: The Role of Supervision in Prevention. American Journal of Lifestyle Medicine. 2010; 4(1): 65–74.

[46] Newman LM, Diekema DS, Shubkin CD, Klein EJ and Quan L. Pediatric wilderness recreational deaths in western Washington State. Ann Emerg Med. 1998(32): 687–692. 9832665 10.1016/s0196-0644(98)70068-x

[47] Wintemute GJ, Anton A, Andrada E, Ribeira R. Compliance with an ordinance requiring the use of personal flotation devices by children in public waterways. West J Emerg Med. 2013(14):200–203. 23599870 10.5811/westjem.2012.1.11717 PMC3628482

[48] Leggat PA, Wilks J. Overseas Visitor Deaths in Australia, 2001 to 2003. Journal of Travel Medicine. 2009; 16(4):243–247. 10.1111/j.1708-8305.2009.00302.x 19674263

[49] Australian Water Safety Council. Australian Rural and Remote Water Safety Plan 2010 to 2015. Australian Water Safety Council. 2010. [cited: 7-07-2016]. Available: http://www.royallifesaving.com.au/__data/assets/pdf_file/0012/4044/Rural_and_Remote_Plan_Final_2010.pdf

[50] Mitchell R, Haddrill K. From the bush to the beach: water safety in rural and remote New South Wales. Australian Journal of Rural Health. 2004; 12(6): 246–250. 15615576 10.1111/j.1440-1854.2004.00628.x

[51] Beattie N, Shaw P, Larson A. Water safety in the bush: strategies for addressing training needs in remote areas. Rural and Remote Health. 2008; 8(2): 855 [cited: 7-07-2016]. Available: http://www.rrh.org.au/publishedarticles/article_print_855.pdf 18498202

[52] Liu H, Laba T-L, Massi L, Jan S, Usherwood T, Patel A, et al Facilitators and barriers to implementation of a pragmatic clinical trial in Aboriginal health services. Medical Journal of Australia. 2013; 203(1):24–28. 10.5694/mja14.00581 26126563

[53] Marchant J, Cheng NG, Lam LT, Fahy FS, Sounndapound SV, Cass DT, et al Bystander basic life support: an important link in the chain of survival for children suffering a drowning or near-drowning episode. Medical Journal of Australia. 2008(188):484–485. 10.5694/j.1326-5377.2008.tb01725.x 18429720

[54] Claesson A, Svensson L, Silfverstolpe J, Herlitz J. Characteristics and outcome among patients suffering out-of-hospital cardiac arrest due to drowning. Resuscitation. 2008; 76(3):381–387. 17997210 10.1016/j.resuscitation.2007.09.003

